# A Comparison of Observational Studies on Subfoveal Choroidal Thickness Measured with OCT according to the Level of Thyroid Eye Disease Activity—Systematic Review and Meta-Analysis

**DOI:** 10.3390/jcm12144720

**Published:** 2023-07-17

**Authors:** Małgorzata Kowalik-Jagodzińska, Maria Sobol, Anna Turno-Kręcicka

**Affiliations:** 1 Department of Ophthalmology, Wroclaw Medical University, 50-367 Wrocław, Poland; anna.turno-krecicka@umw.edu.pl; 2 Department of Biophysics Physiology and Pathophysiology, Medical University of Warsaw, 02-004 Warszawa, Poland; maria.sobol@wum.edu.pl

**Keywords:** thyroid-associated orbitopathy, Graves’ ophthalmopathy, thyroid eye disease, Graves’ orbitopathy, choroidal thickness, optical coherence tomography (OCT)

## Abstract

This study aims to systematise subfoveal choroidal thickness (SFCT) measured using optical coherence tomography (OCT) in patients with different severities of thyroid eye disease (TED) compared with healthy subjects. The PubMed, Web of Science and Scopus databases were searched for the following terms: ((Graves’ ophthalmopathy) OR (thyroid eye disease) OR (Graves’ orbitopathy) OR (thyroid-associated orbitopathy)) AND (choroidal thickness) AND ((optical coherence tomography) OR (OCT)). The pool of papers was narrowed down to articles published until 31 January 2023 (26, 26 and 96 papers, respectively). Twenty-five (25) articles were taken into consideration, which were original papers and included the choroidal thickness measurements among TED patients in their results. Finally, eight papers were included in the comparative analysis of the SFCT parameter in TED patients and a group of healthy controls, and seven papers in the comparative analysis of the same parameter between active and inactive TED patients. The mean value of the difference between the TED group and the healthy group was 38.79 μm, with a confidence interval (CI) from 0.09 to 77.49 μm (*p* = 0.0495). The mean difference between the active TED group and inactive TED group was 38.02 μm, with a CI from 8.62 to 67.42 μm (*p* = 0.0113). All the results were statistically significant.

## 1. Introduction

Thyroid eye disease (TED) is an inflammatory condition of the soft tissues of the orbit which is dependent on the presence of TRAb antibodies in the blood. It is most often one of the symptoms of Graves’ disease, but it is also sometimes diagnosed in the course of other thyroid conditions, e.g., Hashimoto’s disease [1] or in individuals without obvious thyroid dysfunction [2]. For this reason, it is increasingly more common for the orbital disorder to be referred to as ophthalmopathy in the course of thyroid diseases rather than Graves’ disease. TED manifests itself irrespective of the patient’s hormonal status, i.e., in hyperthyroidism, euthyroidism and even hypothyroidism. It is suspected that in cases with undetected levels of TRAb antibodies, their concentration is too low to allow them to be detected with standard laboratory procedures [3]. TED always starts with an acute phase, which can last up to approximately two years [4]. This disorder is usually bilateral; however, exophthalmos may show slight asymmetry and the bilateral nature of the process is only visible in imaging examinations.

The basis for the onset of TED is inflammation mediated by activated T lymphocytes, which, by producing specific pro-inflammatory cytokines (i.e., TNF-alpha, TGF-beta, IL-6, IL-12, IL-17), cause infiltration of the orbital fibroblasts [4,5,6]. Moreover, as a result of interaction with T lymphocytes, B lymphocytes produce antibodies that bind to receptors within the fibroblasts. As a result, these cells differentiate into myofibroblasts and adipocytes, resulting in a pathological increase in tissue volume. Additionally, rising levels of proinflammatory cytokines result in the changed distribution of vasoconstrictive or vasodilative agents with dominancy of the second ones [7]. Knowledge of the molecular basis of inflammation gave rise to targeted anti-inflammatory therapies such as biologic therapies [5,8].

Inflammation of the orbital tissues (mainly the rectus muscles and adipose tissue) causes exophthalmos and abnormal ocular motility as well as congestion and swelling of the ocular adnexae (eyelids, conjunctivae) due to impaired blood outflow from the organ of vision caused by pressure. The characteristic features are swelling and redness of the lacrimal caruncle or corkscrew dilated episcleral vessels. Double vision (diplopia) in binocular vision is a common symptom, the severity of which depends on the level of abnormalities in ocular motility and ocular symmetry. Diplopia may be transient and manifest only in selected gaze directions depending on the severity of inflammatory involvement of individual muscles, or may be a permanent symptom that significantly impairs patients’ quality of life. The most serious complications of orbital tissue inflammation in the course of the condition include exposure keratopathy and neuropathy involving the optic nerve [9]. The mechanism could be compressive, ischaemic or followed by rising intraocular pressure [10].

Magnetic resonance imaging of the orbits shows enlargement of the rectus muscles with characteristic sparing of the tendons, resulting in spindle-shaped muscles, and increased volume and signal from the intraorbital fat. Moreover, the upper eyelid levator muscle or the lacrimal gland may become inflamed [2]. Enlarged tissues reduce the blood flow to the organ of vision through pressure on the vessels that nourish the optic nerve and retina, which can be observed during imaging examinations such as OCT or Doppler ultrasound [10]. Detecting such deviations imperceptible for the patient could accelerate the decision for anti-inflammatory treatment or even surgical decompression.

This study aims to investigate whether subfoveal choroidal thickness (SFCT) is affected by the course of the disease (in the form of a comparison of the SFCT parameter between TED patients and healthy controls) and, additionally, whether the results of SFCT are statistically significantly different in terms of patients with active versus inactive forms of the disease.

## 2. Materials and Methods

**Figure 1:**
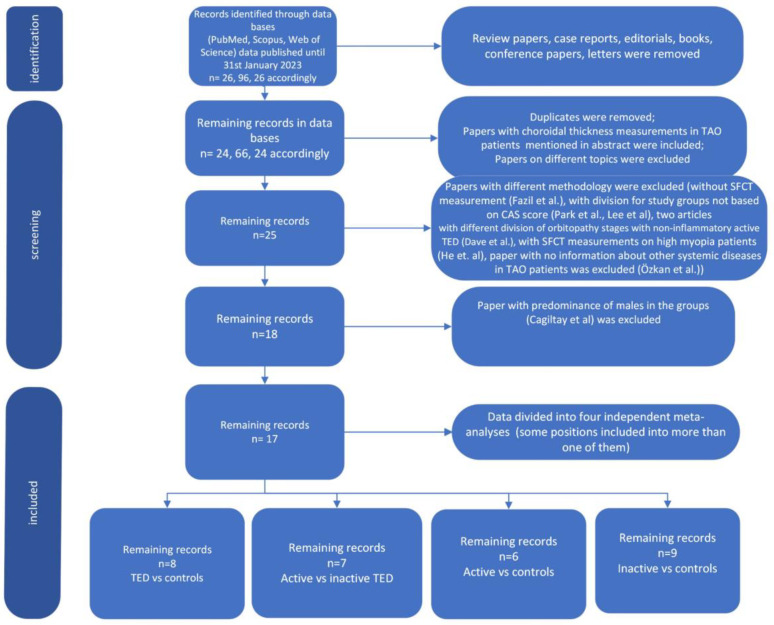
Study selection flow diagram.

Databases such as PubMed, Scopus and Web of Science were used for the analysis. The screening of the results was based on the phrases: (Graves’ ophthalmopathy) OR (thyroid eye disease) OR (Graves’ orbitopathy) OR (thyroid-associated orbitopathy) AND (choroidal thickness) AND (optical coherence tomography) OR (OCT). The pool of papers was narrowed down to articles published until 31 January 2023 (26, 26 and 96 papers, respectively). Twenty-five (25) articles were taken into consideration after repetitive papers were eliminated. The papers considered were original articles and included the choroidal thickness measurements among TED patients in their results. The meta-analysis was conducted in two steps. At the beginning, we presented a comparison of SFCT results in a group of TED patients compared with healthy controls. Then, we extended our analysis to differentiating the SFCT parameter in groups with different levels of TED activity. Some papers were part of more than one analysis. The size of the study was limited due to methodological discrepancies. Studies in which results were reported as median and range; median, range and interquartile range; or mean value but without standard deviation were not included in the analysis. The flow diagram describes the study selection method (Figure 1).

Finally, 8 papers were included in the comparative analysis of the SFCT parameter in TED patients and a group of healthy controls and 7 papers in the comparative analysis of the same parameter between active TED patients and inactive TED patients. Additionally, we conducted two more analyses between active TED and control groups (6 papers) and between inactive TED and control groups (9 papers). SD-OCT, HD OCT or OCT-EDI techniques were used in the studies, while the activity-based division of TED was based on the Clinical Activity Score (CAS) scale.

The systematic review was conducted in accordance with PRISMA guidelines [11]. The detailed requirements are listed below:

Inclusion criteria: original, prospective or retrospective papers based on OCT (SD, HD, SS OCT) in which the SFCT parameter was measured. There was no limit applied to the language of publication. The activity-based division of TED was based on the CAS scale. Adults with diagnosed ophthalmopathy in the course of Graves’ disease or other thyroid conditions were selected for the study group, and healthy subjects of similar age and sex distribution were selected for the control group. 

Exclusion criteria: article types such as case reports, reviews, editorials, letters, books and conference papers. Studies in which results were reported as median and range; median, range and interquartile range; or mean value but without standard deviation. Papers with predominance of males in the groups. SFCT measured on high-myopic patients (values greater that -6 D spheric). Papers with division of active TED for inflammatory and non-inflammatory; other systemic diseases (except arterial hypertension in the paper written by Caliskan et al.); most ophthalmic disorders which could influence the choroid status; surgical and laser therapies in the early past or in general; anti-inflammatory treatment (especially actual corticosteroid therapy); iodine therapy; and poor image quality. In papers with unavailable refraction ranges, inclusion was possible, while no significant differences between study and control groups according to refraction level were described. 

Timing: studies published until 31 January 2023.

### 2.1. Data Extraction

The following data were extracted from each publication: authors, year of publication, study site, number of participants, entry age, sex and subfoveal choroidal thickness. The characteristics of the included studies are summarised in Table 1 and Table 2. Seven studies were from Turkey [12,13,14,15,16,17,18], four from China [19,20,21,22], four from Italy [23,24,25,26] and one from Korea [27].

### 2.2. Statistical Analysis

The statistical analysis was performed using Statistica 13 (Dell Software Inc., Round Rock, TX, USA). The Q test was used to test heterogeneity, and I2 statistics were calculated to quantify and evaluate the heterogeneity (low: 25–50%, moderate: 50–75% and high: >75%). As heterogeneity (I2 statistics) exceeded 97% (study group vs. control group), 88% (active group vs. control group), 88% (inactive group vs. control group) and 92% (active group vs. inactive group), the analysis was performed using a random effects model, and the standardised mean and mean differences were given with a 95% confidence interval (95% CI). Forest plots were generated to describe the difference between groups for SPCT parameter and corresponding 95% confidence intervals (CIs) for each study and overall estimates. To assess the stability of the plotted results, sensitivity analysis was conducted by excluding each study at a time. To assess the publication bias, Egger’s test and Begg’s test were also conducted. The subgroup analysis was conducted to find possible reason of high heterogeneity level. Moreover, the trim-and-fill method for publication bias was performed to estimate potentially missing studies.

## 3. Results

After searching PubMed, Scopus and Web of Science databases, eight eligible publications were included in the comparative analysis of the SFCT parameter in TED and healthy groups, and seven papers in the comparative analysis of the SFCT parameter between active TED patients and inactive TED patients. In the meta-analysis on the comparison of SFCT values between the TED group and the healthy group, 926 eyes were included (including 489 eyes in the TED group and 437 in the control group) at an age range of 19–74 years, respectively, while in the analysis of active TED patients and inactive TED patients compared with the healthy group, 379 eyes (136 in the active TED group, 243 in the healthy group) and 819 eyes (383 in the inactive TED group, 436 in the healthy group), respectively, were included at an age range of 18–65 years. Papers with no comparison results of the TED group with the control group were excluded from the first part of the meta-analysis. Papers with no SFCT measurements for the active and inactive TED groups were excluded from the second part of the conducted meta-analysis. 

The papers included in the analysis differed in the way the eyes were selected and the activity of the condition. The papers by the authors such as Zhang et al., Bruscolini et al., Casini et al., Zhu et al., Lai et al., Del Noce et al., Kurt et al., Caliskan et al. and Loiudice et al. considered both eyes of patients in the control and study groups. The papers by Yeter et al., Gul et al., Ceylanoglu et al. and Yu Lanchu et al. analysed parameters for the right eyes of participants. In the paper by Yildirim et al., the eye with a higher Hertel exophthalmometer measurement score in the exophthalmos group and with more advanced TED according to EUGOGO score in the non-exophthalmos group was selected (if those parameters did not differ between eyes, the eye with the better quality of OCT scans was selected or, at similar scan qualities, the eyes were randomised). Another aspect that differs across the papers included in the analysis is the level of TED activity. In the papers by Zhang et al., Casini et al., Lai et al., Del Noce et al. and Karabulut et al., the study group consisted of TED patients without differentiating the level of TED activity. The papers by Yeter et al. and Bruscolini et al. analysed the comparison between the results of TED patients and the results of healthy controls, and then the study group was divided into the TED active group and the inactive TED group. Yildirim et al. divided the group of TED patients into the exophthalmos group and non-exophthalmos group compared with healthy participants. However, they did not maintain that division when they compared the active TED group with the inactive TED group. Zhu et al., Yu L. et al. and Caliskan et al. compared both the results of active TED patients and inactive TED patients with healthy controls. Loiudice et al. presented a comparison of the results between TED patients and healthy controls along with a comparison of the results for the division of patients according to the form of the disease (active/inactive). Moreover, the result of the SFCT parameter was given for selected patients from the study group with active TED. In the papers by Kim et al., Kurt et al. and Ceylanoglu et al., the study group consisted of inactive TED patients, and active TED was an exclusion criterion for the study group.

**Figure 2:**
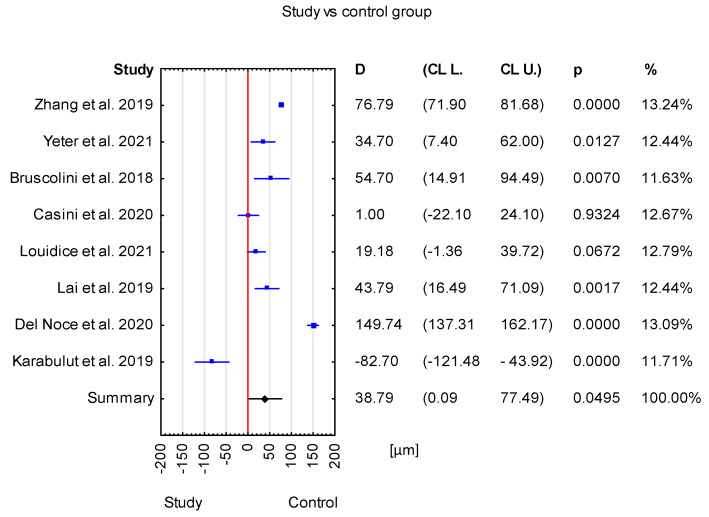
Forest plot of mean difference of SFCT between TED group and control group, *p* value indicating level of statistical significance. The size of the box represents the point estimate for each study in the forest plot and is proportional to that study’s weight-estimate contribution to the summary estimate. Horizontal lines represent 95% CL [13,14,19,20,23,24,25,26].

The mean difference between the TED group and the healthy group was 38.79 μm with a CI of 0.09 to 77.49 μm. This result is statistically significant (*p* = 0.0495) (Figure 2).

**Figure 3:**
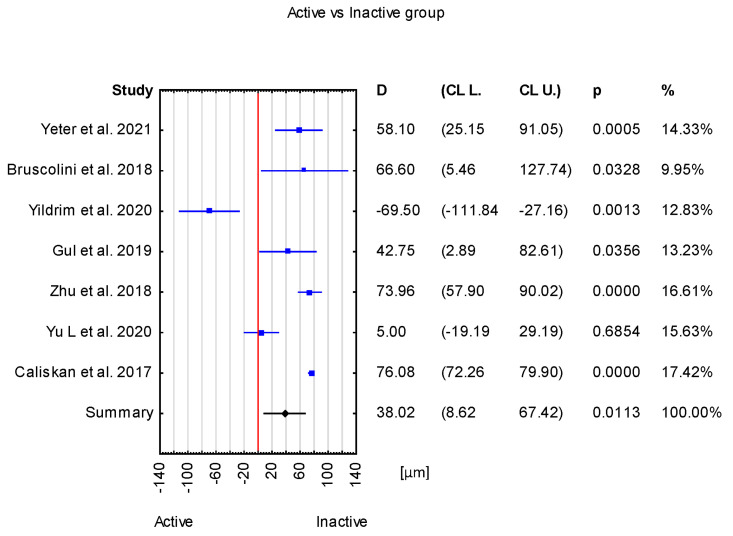
Forest plot of mean difference of SFCT between active and inactive group, *p* value indicating level of statistical significance. The size of the box represents the point estimate for each study in the forest plot and is proportional to that study’s weight-estimate contribution to the summary estimate. Horizontal lines represent 95% CL [12,13,17,18,21,22,23].

The mean difference between the active TED group and inactive TED group was 38.02 μm with a CI of 8.62 to 67.42 μm. This result is statistically significant (*p* = 0.0113) (Figure 3).

**Figure 4:**
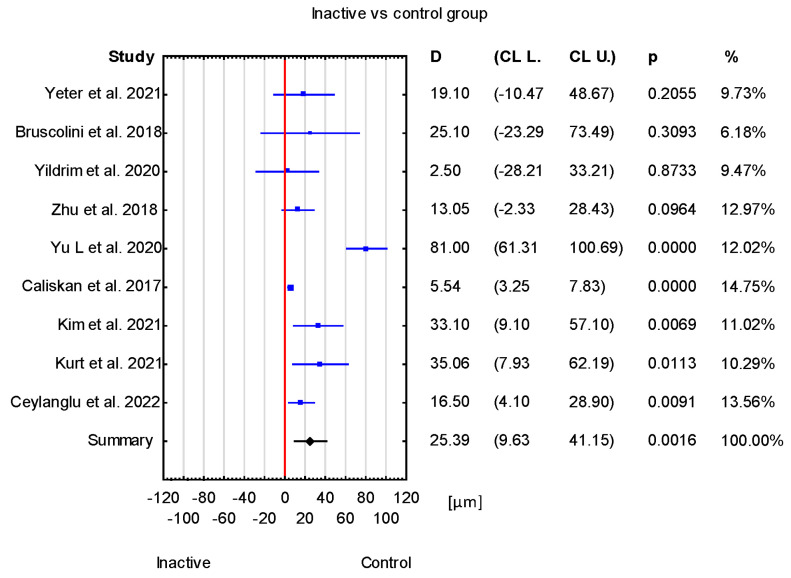
Forest plot of mean difference of SFCT between inactive TED group and control group, *p* value indicating level of statistical significance. The size of the box represents the point estimate for each study in the forest plot and is proportional to that study’s weight-estimate contribution to the summary estimate. Horizontal lines represent 95% CL [13,15,16,17,18,21,22,23,27].

The obtained mean difference between the inactive TED group and the healthy group was 25.39 μm with a CI of 9.63 to 41.15 μm. This result is statistically significant (*p* = 0.0016) (Figure 4).

**Figure 5:**
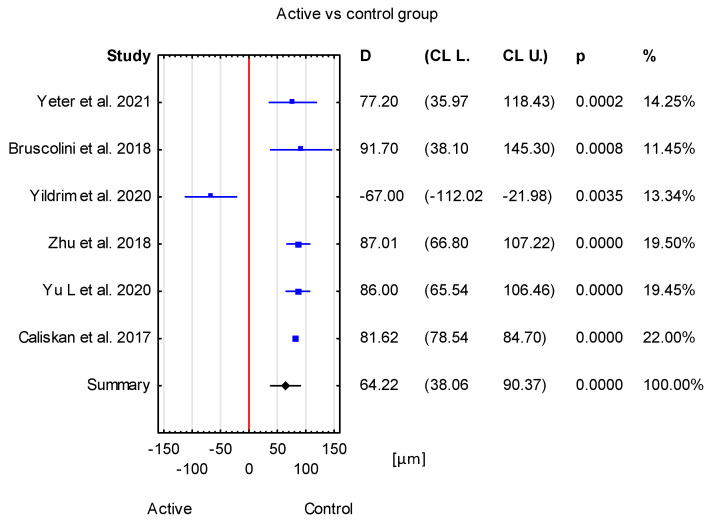
Forest plot of mean difference of SFCT between active TED group and control group, *p* value indicating level of statistical significance. The size of the box represents the point estimate for each study in the forest plot and is proportional to that study’s weight-estimate contribution to the summary estimate. Horizontal lines represent 95% CL [13,17,18,21,22,23].

The obtained mean difference between the active TED group and the healthy group was 64.22 μm with a CI of 38.06 to 90.37 μm. This result is statistically significant (*p* < 0.001) (Figure 5).

**Figure 6:**
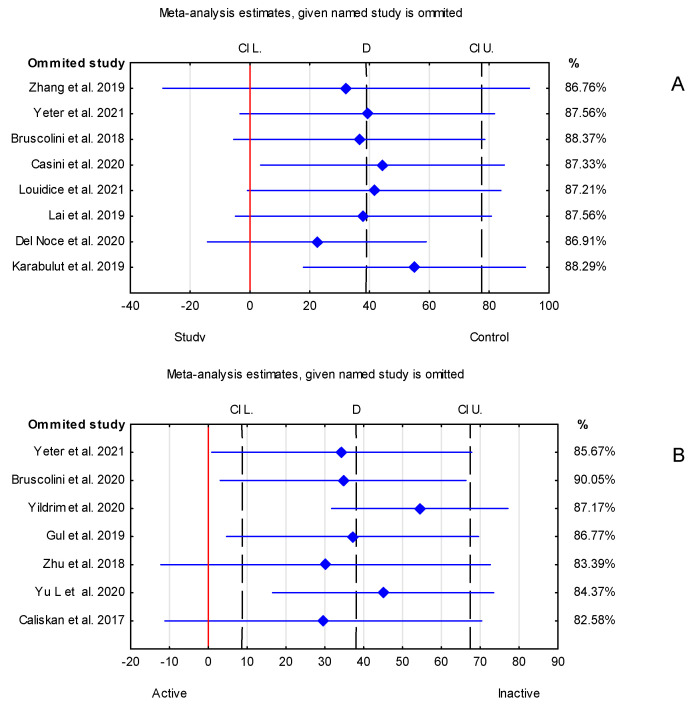

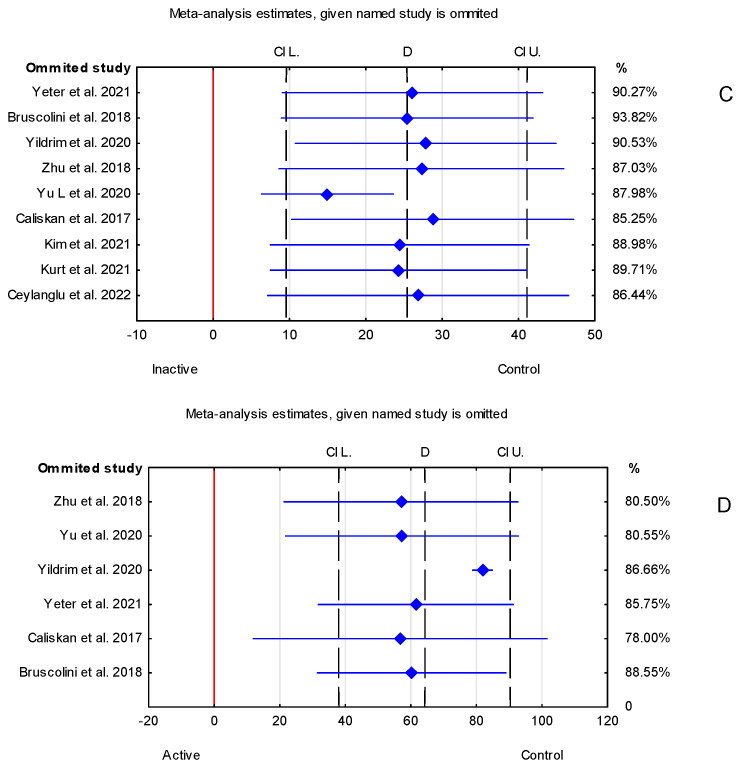
Sensitivity analysis for the effect of individual studies (given named study in the Y axis is omitted) on the pooled difference of SFCT parameter. CI (confidence interval). (**A**) Control vs. study group, (**B**) active vs. inactive group, (**C**) inactive vs. control group, (**D**) active vs. control group [12,13,14,15,16,17,18,19,20,21,22,23,24,25,26,27].

Overall, in the forest plot for the control group vs. study group, the studies denoted higher values of the SFCT parameter for the control group. However, there are differences between the included studies. Studies have Cls of different widths. Two studies, Loiudice et al. [25] and Casini et al. [24], include the value of zero (no effect), while others do not. Moreover, the study by Casini et al. [24] has point estimates that even fall right on the no-effect line, and the study by Karabulut et al. [14] estimates even lower values of SFCT for the control than the study group. Similarly, in the case of active vs. inactive groups, CIs of different widths can be noticed, and in the study by Yıldırım et al. [17], the active-group value of SFCT is lower than that in the inactive group. In the case of active vs. control groups, the CIs are different widths, and in Yıldırım et al. [17], the SFCT value is lower for the active group compared with the control group. For inactive vs. control groups, the value of zero (no effect) can be noticed in four studies [13,17,22,23] (Figure 6). 

An Egger’s publication bias was generated. The results of the Egger’s test (*p* = 0.257 for control vs. study group, *p* = 0.080 active vs. inactive, *p* = 0.060 inactive vs. control and *p* = 0.445 active vs. control) indicated that there were minimal potential risks of publication bias, which was also consistent with the Begg’s test.

### 3.1. The Subgroup Analysis

**Figure 7:**
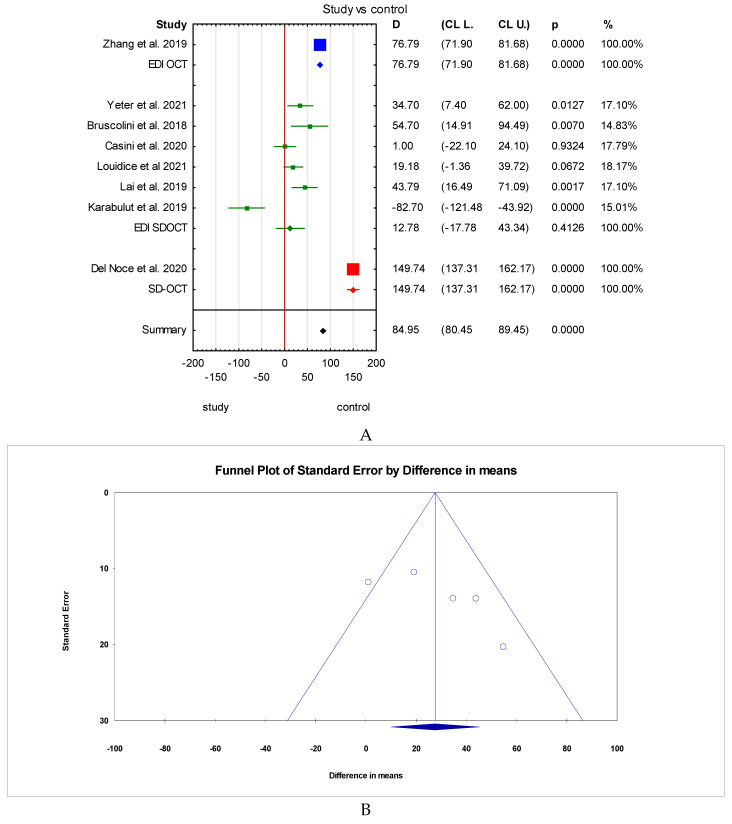

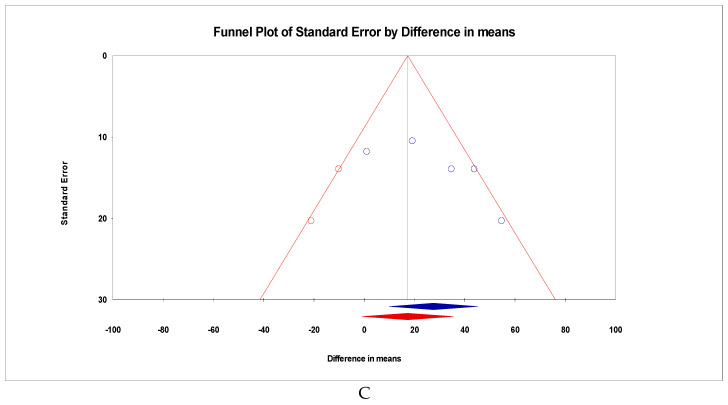
(**A**) Subgroup analysis. Funnel plots before (**B**) and after (**C**) applying the trim-and-fill method, imputed missing studies (red dots) [13,14,19,20,23,24,25,26].

Since the heterogeneity among studies was high, a subgroup analysis for study vs. control group was performed to try to find possible reasons for the variability of the results. The method of measuring OCT was considered for the subgroup analysis. Six publications [13,14,19,23,24,25] which were included in the meta-analysis used the EDI-SDOCT method, whereas one study [26] used SD-OCT with no EDI mode mentioned. Furthermore, although Zhang et al. [20] stated that they used the EDI-OCT method, they did not specify whether it was SD or HD OCT. In the subgroup containing six publications which all used the EDI-SDOCT method, the overall result was statistically insignificant. Statistically significant results were received for the two subgroups containing Zhang et al. and Del Noce et al. individually (Figure 7A). The subgroup analysis can suggest that the method of OCT measurement impacted the overall meta-analysis results. However, it is important to note that two of the subgroup analyses contained only one study; thus, the true effect is difficult to establish.

### 3.2. The Trim-and-Fill Method

The trim-and-fill method was used to estimate the effect of potentially missing studies due to publication bias in the funnel plot. After trimming the studies that caused the funnel plot’s asymmetry [14,20,26], the SFCT result was 27.55 μm (95% confidence interval from 13.26 to 36.26 μm). Using trim-and-fill, the imputed point estimate was 17.29 μm (95% confidence interval from −1.02 μm to 35.60 μm) (Figure 7B,C).

## 4. Discussion

To the best of our knowledge, this systematic review using meta-analysis is the first to provide a comprehensive overview of the subfoveal choroidal thickness (SFCT) measured using optical coherence tomography (OCT) in patients with different severities of thyroid eye disease (TED). Because of the high availability of repeatable and easy OCT examination, measuring the SFCT parameter among the population with thyroid disorders could be a relevant option to diagnose early stages of orbital involvement.

Ocular blood flow in TED has long been of interest in research, and was initially assessed using Doppler ultrasound [28,29,30,31]. Nevertheless, this method is an examination that is strictly dependent on the investigator’s skills and is much less accessible than OCT. OCT allows imaging of all layers of the retina and choroid, which is the main source of blood and nutrients for the retina. Moreover, the possibility of using Angio-OCT provides further scope to accurately visualise the quality of the central retinal vascularity and vascularity of the optic nerve in the TED in question. Monitoring changes in blood flow using OCT—due to the ease with which the examination can be performed and the possibility of remembering the imaging point for each patient in the form of selecting an examination with follow-up—is associated with lower measurement error [32].

The earliest published study showing differences in the SFCT parameter of TED patients compared with healthy controls was the 2016 study by Ozkan et al. [33]. The study was conducted on a group of TED patients and healthy controls. All the participants underwent ophthalmic examination with EDI-OCT examination with SFCT assessment. Additionally, VEP and perimetric examinations were performed. There is a first observation described in the conclusion that SFCT is thicker in GO patients than in healthy controls, and the parameter correlates with CAS and VEP P100 latency. The paper was not enrolled in our analysis because of insufficient description of other systemic disorders of GO patients. The following study assessing SFCT in GO was the 2017 study by Caliskan et al. [18]. The study was conducted in 2015–2016 on patients with confirmed Graves’ ophthalmopathy, without comorbid diseases apart from hypertension. Patients who had undergone antithyroid treatment, radioiodine therapy or thyroidectomy for the past three months were excluded. Also excluded were patients with ophthalmological diseases that might affect the retina (glaucoma, AMD, diabetic retinopathy, inflammatory diseases of the uvea) and patients with diagnosed optic neuropathy. The appropriate quality of OCT scans was also a precondition for qualifying patient results. A division was made between active TED patients and inactive TED patients, and the results were compared with a similarly sized group of patients without comorbid diseases. Results of both eyes of the study participants were included in the analysis. The age and sex distribution in the study and control groups was similar. The duration of TED was 5.0 ± 7.1 years in the active TED group and 6.20 ± 4.2 years in the inactive TED group. The SFCT parameter was proven to be higher in both active and inactive TED groups compared with healthy controls; however, the difference was significantly greater for those with a CAS score ≥ 3. Moreover, there a statistically significant correlation was found between SFCT and disease duration, intraocular pressure, exophthalmos size and thyroid function.

However, no pooled analysis was performed for TED patients regardless of activity level compared with healthy controls. There is also no information on the known factors that affect variation in choroidal thickness, such as circadian rhythm or pregnancy and breastfeeding status, which—given the preponderance of women (also of childbearing age)—may affect the results of the analysis [34,35].

Studies published in later years paid much more attention to the time of the imaging examination and the pregnancy status. Moreover, the size of the refractive error was added to the exclusion criteria of the study.

The SFCT parameter was analysed mainly in correlation with CAS, EUGOGO classification, proptosis, IOP, TRAb and free thyroid hormones level. Overall, SFCT was positively correlated with the aforementioned parameters, but statistical significancy differed among the studies. For instance, Zhang et al. proved that SFCT positively correlated with IOP, exophthalmometric measurements and LCVT, but the differences were not statistically significant. Kurt et al. and Zhu et al. described positive significant correlation between SFCT and CAS; SFCT was thicker in the inactive TED group than in the control group in the first study and SFCT was also thicker in the active TED group than in the inactive and control groups in the second study. In the paper of Kim et al., we can find the information that age, axial length and exophthalmos level significantly affected the SFCT, contrary to CAS, which has no significant influence on SFCT measurement. What is interesting is that Lai et al. have additionally proven that thinner SFCT in TAO patients relates to poorer visual acuity, greater exophthalmos and is seen among older patients and longer eye bulbs. Ceylanoglu et al. also found an association between SFCT and age and duration of the disease, but no correlation was detected between the parameter and gender, habit of smoking, TRAb level and selenium administration. No correlation was found between SFCT and the habit of smoking, diplopia or the severity grading of GO in the Bruscolini et al. paper. Similarly, no association with the EUGOGO classification was found in the Yildirim et al. study. In opposition were Del Noce et al., whose results showed a positive correlation between SFCT and the EUGOGO classification. Negative correlation was found in Bruscolini et al.’s study between SFCT and disease duration. Caliskan et al. presented that thicker SFCT was significantly associated with higher CAS, longer duration of Graves’ disease, higher level of thyroid hormones, higher IOP and greater proptosis, but not significantly associated with sex, treatment for GD, best-corrected visual acuity and axial length. 

The first systematic review that shows, among other things, a comparison of the results of the SFCT parameter in TED patients is a paper published in November 2022 by Chien et al. [35]. In this study, in addition to parameters describing the thickness of the uvea at different measurement points, retinal blood flow parameters in the macular area were also analysed using Angio-OCT. A meta-analysis was not attempted because there was little homogeneity in the measurement methodology using the Angio-OCT machine, which prevented a reliable comparison. In terms of SFCT comparisons, this review presents papers that also differ from one another according to methodology. With such a large number of studies listed in the paper, the analysis would have resulted in highly inaccurate results. 

Goel et al. published a systematic review in January 2023, which presented the aspect of orbital and eye perfusion according to colour Doppler imaging, OCTA and OCT images of the macular region in a TED population [10]. The part considering strictly the SFCT parameter involved 18 original papers. The majority of publications show typical thickening of the choroid in TED patients, especially in their active phase. The authors explained that thinning of the choroid during TED course, as it was observed in few publications, may be the consequence of homeostatic processes to keep the bulbar perfusion or may be the paradoxical sign of compression on the eye bulb during the active phase of the disorder.

The conducted meta-analyses were based on data from 17 articles (8 in the control vs. study group analysis, 7 in the active vs. inactive analysis, 6 articles in the active vs. controls and 7 articles in the inactive vs. controls analysis) chosen from 24 preselected positions. Publications which presented only the result for the active TED group and inactive TED group without the overall result for both groups compared with the control group were not taken into consideration. It would result in taking the healthy controls into account two times, and thus distorting the final result. Similarly, a study that divided the active TED group into the exophthalmos group and non-exophthalmos group was excluded from the analysis due to the lack of a total result for active TED [17]. Also, the meta-analysis of the SFCT parameter between the active and inactive TED groups did not include the data presented by Loiudice et al. because that paper only reported total results for the study group, of which the result for active TED patients was extracted without reporting the results for the rest of the study group [25]. Both papers by Dave et al. were excluded because the results were too similar, which would have significantly distorted the quantitative proportions and posed a risk of multiplying the results [36,37]. Furthermore, the authors included the concept of non-inflammatory active TED, which does not appear in any other publication. Another excluded paper is the study by Cagiltay et al. due to the fact that the authors’ ratio of women to men that qualified for the study differed significantly from that of the other papers [38,39]. The Turkish paper written by Fazil et al. was excluded because of using an average CT measurement instead of SFCT [40], and the Chinese research conducted by Yu Nan et al. [41] was excluded due to the higher myopic level of the participants.

The resulting large value of coefficient I2 may result from the fact that the meta-analyses included the papers by Del Noce et al., Casini et al. and Karabuulut et al., whose results differ significantly from those presented in the other studies. In the study by Del Noce et al., the SFCT value for the control group was 135.89, which is quite low and may be explained by the fact that this is the study with the lowest mean age of participants [42]. After an analysis of the group characteristics, it can be found that the oldest patient was 49 years old, which is a lower age than in the other studies. In contrast, the study by Karabuulut et al. is the only one in which the control group scored higher than the study group (396.3 vs. 313.6), while in addition to the measurement in the centre of the macula itself, the choroidal thickness was measured in the temporal part and nasal part from the fovea, and these results were already higher in the study group than in the control group. As the normal choroid is the thickest in the centre of the fovea, chronic compression with atrophic changes in the macular region could be the explanation of such a discrepancy [43].

Limitations that may affect the final outcome of the present meta-analyses are the heterogeneous duration of Graves’–Basedow disease (from 1 month [17] to almost 19 years [15]), the duration of the ocular involvement (the average time was mentioned from circa 2 years [16] to 6 years [18]) or the lack of mention of this topic in some of the publications [12,13,14,24,25,26,27]. In papers published in Chinese or Korean, the aspect of disease duration was not available due to language limitations [20,22]. Furthermore, studies reported the duration of TED or Graves’ disease, which is not the same and may interfere with the correct interpretation of the final results. Another aspect is the ethnicity of participants (Italian, Turkish, Asian studies), which is connected with different anatomy of the orbits and the wide disparity in the age ranges of the groups of participants among the publications analysed. Nevertheless, an important confounding factor is the different way in which different authors select eyes for analysis—qualifying both eyes of the participant or selecting one of them. The sex distribution was remarkably similar between the studies selected for the final analysis, with a preponderance of women. Smoking status and BMI measurements should have been taken into consideration during planning the inclusion and exclusion criteria of participants in the research because they may significantly influenced the outcomes [44,45]. Technical issues, such us different OCT techniques and mainly manual SFCT measurements using calipers that depend on the researcher skills, can also cause a large discrepancy in the results. The one study led by Kurt used the SS-OCT technique with automatic measurement of the parameter. Figure 6 demonstrates that omitting this paper has no significant impact on the overall active vs. inactive group analysis.

Moreover, as there are no data including division according to gender, age, duration of disease or use of steroid in the available literature, we could not divide groups into subgroups to discover potential sources of heterogeneity.

More studies that include the TED duration parameter are needed to reduce the heterogeneity of results in future meta-analyses concerning the SFCT parameter. It would be beneficial to conduct studies at the same time of the day and rely on papers using eye randomisation in participants. What is more, longitudinal studies on TED groups with SFCT measurements before and after medical intervention (glucocorticoid treatment, radioiodine therapy, biological agents) could bring beneficial impacts.

## 5. Conclusions

This study showed statistically significant differences in terms of the SFCT parameter between the study and control groups and active and inactive forms of TED. This brings the opportunity to use OCT in TED patients to assess retinal blood flow abnormalities resulting from the severity of the condition. All authors have read and agreed to the published version of the manuscript.

## Figures and Tables

**Table 1:** Characterisation of publications presenting SFCT parameter differences between TED and healthy subjects.

Publication Title	Quantity of TED Group, F/M	Quantity of Control Group, F/M	Age of TED Group (Average ± SD)	Age of Control Group (Average ± SD)	SFCT in TED Group	SFCT in Control Group	OCT Method
Zhang, Dan Zhao, Jun Zhang, Juan Mei et al., Analysis of different choroidal stratification thickness under macular fovea in patients with early graves ophthalmopathy [20]	31 (62 eyes)	31 (62 eyes)	37 1 ± 12 5 (19–58)	Age-matched to the study group	315.79 ± 14.99 μm	239.00 ± 12.67 μm	EDI OCT
Volkan Yeter, Nurullah Kocak, Mustafa Suba, et al., Choroidal vascularity index in thyroid-associated ophthalmopathy [13]	53 (53 eyes), 30/23	53 (53 eyes), 30/23	46.7 ± 13.3 (19–70)	46.9 ± 13.5 (19–70)	324.0 ± 63.8 (165–461) μm	289.3 ± 78.8 (126–432) μm	EDI SDOCT (Heidelberg)
Bruscolini Alice, La Cava Maurizio, Gharbiya Magda et al., Management of patients with Graves’ disease and orbital involvement: Role of spectral domain optical coherence tomography [23]	18 (36 eyes), 10/8	18 (36 eyes), 11/7	44.1 ± 9.8 (24–57)	44.2 ± 10.7 (26–60)	399.2 ± 84.1 μm	344.5 ± 88.1 μm	EDI SDOCT (Heidelberg Spectralis)
Casini Giamberto, Marinò Michele, Rubino Marisa et al., Retinal, choroidal and optic disc analysis in patients with Graves’ disease with or without orbitopathy [24]	40 (80 eyes), 35/5	40 (80 eyes), 29/11	39.57 ± 5.60 (23–49)	36.28 ± 5.79 (29–49)	288 ± 88 μm	287 ± 58 μm	EDI SDOCT (Heidelberg)
Pasquale Loiudice, Marco Pellegrini, Michele Marinò, et al., Choroidal vascularity index in thyroid-associated ophthalmopathy: a cross-sectional study [25]	40 (80 eyes)	40 (80 eyes)	39.30 ± 4.54 (30–45)	37.45 ± 4.44 (28–45)	308.08 ± 73.37 μm	288.90 ± 58.32 μm	EDI SDOCT (Heidelberg)
Joohyun Kim, Sumin Yoon and Sehyun Baek, Increase of central foveal and temporal choroidal thickness in patients with inactive thyroid eye disease [27]	49 (49 eyes), 34/15	49 (49 eyes), 34/15	51.1 ± 13.2	50.2 ± 14.4	294.2 ± 71.4 μm (only inactive TED)	261.1 ± 47.4 μm	(EDI) mode by cirrus HD-OCT (Carl Zeiss Meditec Inc., Dublin, CA, USA)
Frank H P Lai, Tiara W U Iao, Danny S C Ng, et al., Choroidal thickness in thyroid-associated orbitopathy [19]	52 (104 eyes), 35/17	26 (52 eyes), 16/10	47.4 ± 13.2	45.2 ± 15.6	331.29 ± 83.67 μm	287.50 ± 78.55 μm	EDI SDOCT (Spectralis; Heidelberg)
Chiara Del Noce, Aldo Vagge, Massimo Nicolò, et al., Evaluation of choroidal thickness and choroidal vascular blood flow in patients with thyroid-associated orbitopathy (TAO) using SD-OCT and Angio-OCT [26]	18 (36 eyes), 14/4	18 (36 eyes), 11/7	26.5 ± 4.94 (19–74)	26.5 ± 3.53	285.6275 ± 32.5 μm	135.89 ± 19.8 μm	SD-OCT Topcon ImageNet 6 (DRI OCT Triton, Topcon Corporation).
Gamze Ozturk Karabulut, Korhan Fazil, Can Ozturker, et al., Do ocular pulse amplitude and choroidal thickness change in patients with thyroid eye disease? [14]	38 (38 eyes), 27/11	38 (38 eyes), 22/16	49 ± 11.8	49.07 ± 11.9	313.6 ± 76.1 μm	396.3 ± 95.3 μm	EDI SDOCT, (Spectralis; Heidelberg)
Muhammed M Kurt, Cetin Akpolat, Ferhat Evliyaoglu, et al., Evaluation of Retinal Neurodegeneration and Choroidal Thickness in Patients with Inactive Graves’ Ophthalmopathy [15]	29 (58 eyes), 14/15	30 (60 eyes), 18/12	40.66 ± 11.83	42.60 ± 12.38	305.53 ± 87.93 μm (only inactive TED)	270.4 7 ± 60.35 μm	SS-OCT (automated module of the Triton)
Kubra Serbest Ceylanoglu, Nazan Acar Eser, Emine Malkoc Sen, Choroidal structural evaluation in inactive Graves’ ophthalmopathy [16]	56 (56 eyes), 30/26	64 (64 eyes, 34/30)	39.5 ± 11.4	42.2 ± 5.6	311.9 ± 30.0 μm (only inactive TED)	295.4 ± 38.1 μm	EDI SDOCT Heidelberg Spectralis

**Table 2:** Characterisation of publications qualified for analysis of SFCT parameter differences between active and inactive TED patients.

Publication Title	Quantity of Active TED Group (F/M)	Quantity of Inactive TED Group (F/M)	Age of Active TED Group	Age of Inactive TED Group	SFCT Active TED	SFCT Inactive TED	OCT Method
Volkan Yeter, Nurullah Kocak, Mustafa Suba, et al., Choroidal vascularity index in thyroid-associated ophthalmopathy [13]	17 eyes	36 eyes			366.5 ± 63.6 (247–461) μm	308.4 ± 53.9 (165–396) μm	EDI SDOCT (Heidelberg)
Bruscolini Alice, La Cava Maurizio, Gharbiya Magda et al., Management of patients with Graves’ disease and orbital involvement: Role of spectral domain optical coherence tomography [23]	8 (16 eyes)	10 (20 eyes)			436.2 ± 97.5 μm	369.6 ± 89.3 μm	EDI SDOCT (Heidelberg Spectralis)
Yıldırım Gamze, Şahlı Esra, Alp Mehmet Numan, Evaluation of the effect of proptosis on choroidal thickness in graves’ ophthalmopathy [17]	9 (9 eyes)	41 (41 eyes)			249.1 ± 45.4 μm	318.6 ± 61.0 μm	EDI HD-OCT (Cirrus, Carl Zeiss Meditec Inc., Dublin, CA, USA).
Adem Gul, Ekrem Basural, Hilal Eser Ozturk, Comparison of choroidal thickness in patients with active and stable thyroid eye disease [12]	24 (24 eyes) (15/9)	23 (23 eyes) (19/4)	40.7 ± 11.8 (22–60)	43.7 ± 11.9 (18–56)	304.79 ± 75.19 μm	262.04 ± 63.46 μm	EDI HD-OCT (Cirrus, Carl Zeiss Meditec Inc., Dublin, CA, USA).
Zhu Y, Song Y, Cai Q, et al., A study on observing the central macular choroidal thickness of thyroid-associated ophthalmopathy patients with spectral-domain optical coherence tomography [22]	11 (22 eyes)	21 (42 eyes)			391.27 ± 33.89 μm	317.31 ± 29.62 μm	SD-OCT
Lanchu Yu, Qin Jiao, Yu Cheng, Yanji Zhu, Zhongjing Lin, Xi Shen, Evaluation of retinal and choroidal variations in thyroid-associated ophthalmopathy using optical coherence tomography angiography [21]	20 (20 eyes) (12/8)	33 (33 eyes) (27/6)	43.5 ± 11.5	39.3 ± 11.3	304 ± 41 μm	299 ± 45 μm	HD-OCT (Carl Zeiss Meditec, Dublin, CA, USA). The
Sinan Çalışkan, Mutlu Acar, Canan Gürdal, Choroidal Thickness in Patients with Graves’ Ophthalmopathy [18]	14 (28 eyes, 12/2)	24 (48 eyes, 20/4)	46.7 ± 14.3 (23–65)	40.5 ± 10.1 (25–56)	395.84 ± 9.68 μm	319.76 ± 7.07 μm	SD-OCT (RTVue-XR, software version 6.1, Optovue, Inc., Fremont, CA, USA)

## Data Availability

All data generated or analyzed during this study are included in this published article. The review was not registered and the review protocol was not prepared.
